# Membrane Fractionation of Protein Hydrolysates from By-Products: Recovery of Valuable Compounds from Spent Yeasts

**DOI:** 10.3390/membranes11010023

**Published:** 2020-12-29

**Authors:** Gabriela Vollet Marson, Marie-Pierre Belleville, Stella Lacour, Miriam Dupas Hubinger

**Affiliations:** 1 Institut Européen des Membranes, IEM—UMR 5635, ENSCM, CNRS, Université de Montpellier, CC 047, 2 Place Eugène Bataillon, 34095 Montpellier CEDEX 5, France; marie-pierre.belleville@umontpellier.fr (M.-P.B.); stella.lacour-cartier@umontpellier.fr (S.L.); 2 Department of Food Engineering, School of Food Engineering, UNICAMP, Rua Monteiro Lobato, 80, Campinas 13083-862, Brazil; mhub@unicamp.br

**Keywords:** *Saccharomyces* sp., protein hydrolysis, membrane separation technology, ultrafiltration, membrane-peptide interactions, spent brewer’s yeast

## Abstract

Spent brewer’s yeast (*Saccharomyces* sp.), the second most generated by-product from the brewing industry, contains bioactive and nutritional compounds with high added value such as proteins (40–50%), polysaccharides, fibers and vitamins. Molecules of interest from agro-industrial by-products need to be extracted, separated, concentrated, and/or purified so that a minimum purity level is achieved, allowing its application. Enzymatic hydrolysis has been successfully used in the production of peptides and protein hydrolysates. The obtained hydrolysates require efficient downstream processes such as membrane technology, which is an important tool for the recovery of thermolabile and sensitive compounds from complex mixtures, with low energy consumption and high specificity. The integration of membrane techniques that promote the separation through sieving and charge-based mechanisms is of great interest to improve the purity of the recovered fractions. This review is specifically addressed to the application of membrane technologies for the recovery of peptides from yeast protein hydrolysates. Fundamental concepts and practical aspects relative to the ultrafiltration of agro-industrial protein hydrolysates will be described. Challenges and perspectives involving the recovery of peptides from yeast protein hydrolysates will be presented and thoroughly discussed.

## 1. Introduction

Membrane separation technologies have been successfully applied and can be considered to be an integral part of the downstream processing of agro-industrial, food, pharmaceutical and biotechnological products. The related industries annually produce huge amounts of by-products that not only have high chemical oxygen demand (COD) but also require proper handling and disposal. For these reasons, these by-products represent a serious economic and environmental concern worldwide. In an attempt to address these issues and to promote a more sustainable industrial production, processing technologies are being developed to foster the re-use and recovery of potential high value-added compounds from those streams [1,2].

Separation processes for the treatment of bio-based by-products demand productive, efficient and sufficiently robust technologies to account for the intrinsic variability and sometimes fluctuating availability of some by-products throughout the year. The treatment of agro-industrial by-products involves an elaborate approach because those materials have a complex composition and a high organic load, thus requiring specific extraction prior to separation [3]. Membrane processing technologies usually offer high throughput associated with very good product purity, thereby allowing an efficient wastewater treatment approach (to produce recycling water) as well as recovery of several valuable by-product components [2,4,5]. With these technologies, one can combine productivity with separation efficiency and reduce the number of processing steps. Different objectives can be achieved, such as clarification, fractionation, purification and concentration [6,7].

The brewing industry produces several tons of spent brewer’s yeast (SBY) per year. As with other bio-based by-products, this residue has a high COD that needs to be managed properly [8]. The potential for re-use and transformation of this material has been addressed by several authors in an attempt to reduce the environmental impact of beer production and to promote the valorization of a nutrient-rich by-product. SBY consists of yeast cells collected after fermentation/maturation of beer and is quite rich in proteins (40–50%, d.w.), carbohydrates, vitamins, minerals and other compounds of interest for the food and pharmaceutical industries, such as β-d-glucans, 5’nucleotides, complex B vitamins and bioactive peptides [9,10,11].

The release of peptides from SBY requires steps such as chemical or mechanical cell wall rupture and proteolytic hydrolysis to ensure cell wall disruption and transformation of proteins into peptides [12]. The resulting yeast extract contains several macro and micronutrients that need to be properly separated before they can be applied as new ingredients. Thus, aiming at a more purified product, with a higher protein content and fewer contaminants, the hydrolysate needs to be treated. Separation and fractionation of yeast proteins can be carried out by chromatographic methods [13,14], which have high selectivity but very high operating costs; moreover, they are not simple to scale up [15].

Sometimes, membrane separation processes are used before enzymatic hydrolysis with the objective of performing hydrolysis of specific fractions. Amorim et al. [16] ultrafiltered (10 kg mol^−1^) an SBY autolysate before enzymatic hydrolysis with a *C. carduculus* extract. After hydrolysis, a 3 kg mol^−1^ ultrafiltration step and reverse osmosis were carried out. Versatile and continuous separation and hydrolysis can be performed simultaneously in enzymatic membrane reactors. This procedure has been used for production of protein hydrolysates from fish, milk and other products [17,18]. Despite these applications, major use of membrane technologies is still in the downstream stages of separation of protein hydrolysates from complex matrices [16,19]. Membrane separation technology has been used to fractionate and concentrate protein hydrolysates of by-products with biological and functional properties [15,20,21,22], including SBY peptides [9,16,23].

Properties of peptides and proteins depend on their sequence and structure. Thus, their separation from mixtures of complex composition must be carried out by mild methods (low temperatures and pH value close to neutrality) with high selectivity, in order to maintain the structural and physicochemical characteristics of molecules, as ensured by many membrane separation technologies [24,25,26]. Separation performance is determined by membrane selectivity and permeate flux, which are dependent on operating conditions (temperature, pressure, process configuration, module characteristics, cleaning procedure), membrane properties (membrane material and structure, membrane pore size) and feed characteristics (pH, concentration, composition and physicochemical characteristics of feed components) [27,28]. The intended purpose of separation is also an important aspect of process design [2].

This review is a state-of-the-art of membrane processes applied to fractionation of protein hydrolysates from agro-industrial by-products, mainly from spent yeasts. Yeast by-products are feeds of very complex composition that are recently being explored as sources of bioactive molecules, with only a few studies published on its separation particularities. The influencing factors and possible separation strategies using membranes are not found in the literature. Thus, the aim of this review is to gather the latest works published in the area and to outline the main strategies and factors to be considered for the treatment of these materials. The first two sections focus on the current strategies, challenges and solutions for the application of pressure-driven membrane technology to the downstream processing of protein hydrolysates. Then, in a third section, the use of charge-based membrane separation techniques is presented. Finally, in the last section, particularities involving the separation of SBY protein hydrolysates are discussed by taking into account engineering, technical and practical aspects of membrane processes.

## 2. Processing Strategies of Protein By-Products Using Pressure-Driven Membrane Operations

**Figure 1:**
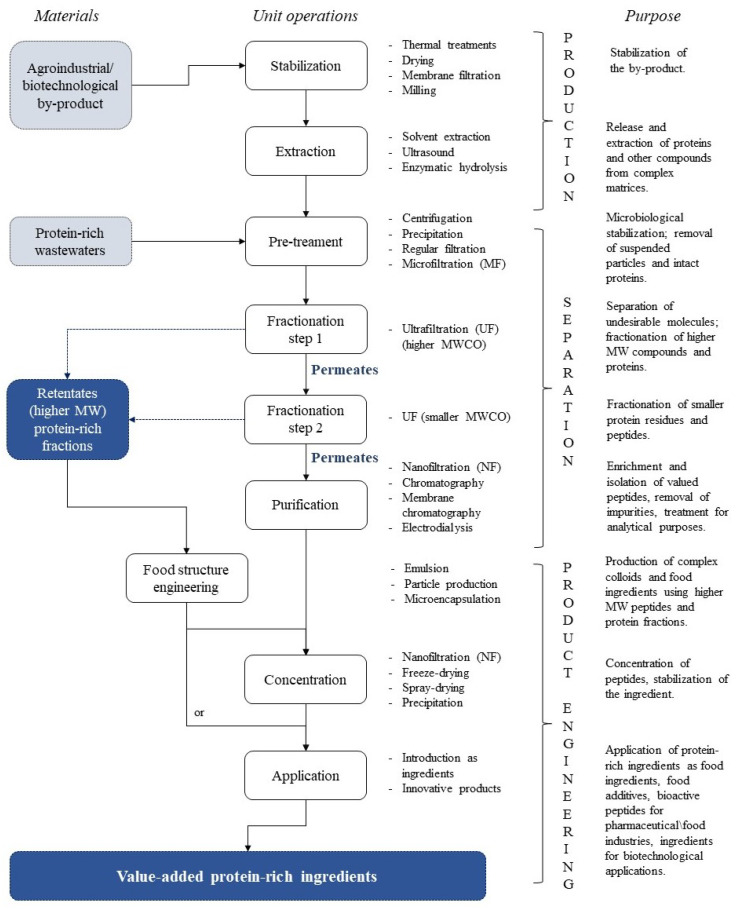
Strategy of processing of agro-industrial, food and biotechnological protein-rich by-products into value-added ingredients.

Figure 1 shows a schematic flow of potential unit operations involved in the transformation of agro-industrial and biotechnological by-products into value-added protein-rich ingredients. The transformation of by-products from biotechnological, food and agro-industrial processing begins with the stabilization of the material. Processing of by-products may not be performed in the same plant where the material is produced. Stabilization avoids any chemical and microbiological degradation of the material while ensuring its safety during proper transport and storage before further processing. Thermal treatments that are able to inactivate endogenous enzymes that may alter the characteristics of the by-products, are usually applied. Other unit operations such as milling, drying, thermal treatments and conventional filtration, either combined or alone, can also be used for this purpose [1].

The next step is to properly release proteins from the original structure of the material. The extraction step is essential in the transformation of by-products because of their complex composition and the usually low initial availability of the compounds of interest. Protein extraction from food and biotechnological matrices is achieved by enzymatic treatments, which offer higher specificity, efficiency and control, not involving the use of toxic chemicals. Chemical extraction methods and ultrasound technology can also be employed. A successful recovery of value-added components from by-products can be achieved when extraction is coupled to efficient downstream separation processes [1]. A complex pool of protein fractions and peptides is obtained in addition to many other original compounds of the material, such as polysaccharides, fibers, minerals, vitamins, nucleic acids, etc. The resulting extract usually needs to be treated to ensure higher performance in the next downstream processing stages. Several technologies are used for this purpose, namely centrifugation, protein precipitation, conventional filtration or use of adsorbents (activated carbon, diatomaceous earth) and MF membranes [3,4]. MF is largely employed to clarify and reduce microbial count and macromolecules such as non-hydrolyzed proteins, lipids, fibers and other aggregates, while retaining suspended colloidal particles produced during fermentation and processing [29]. MF also contributes to the clarification of solutions prior to fractionation steps.

**Figure 2:**
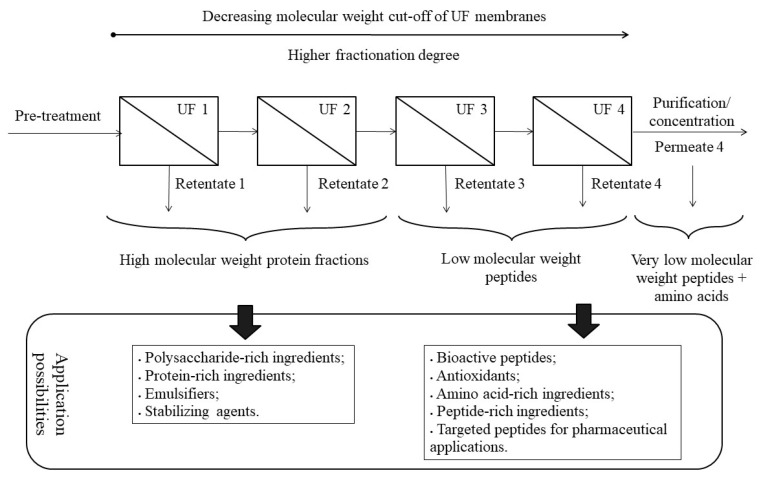
Ultrafiltration (UF) fractionation cascades for the recovery of several protein and peptides-based ingredients, using 500–1 kg mol^−1^ molecular weight cut-off membranes.

The recovery of compounds of interest will require one or more fractionation steps, usually achieved using UF, followed by purification or concentration steps, per requirement of the targeted ingredient, as shown in Figure 2. UF is the main pressure-driven process used in the processing of proteinaceous solutions because UF molecular weight cut-offs (MWCOs) fit the size range of proteins and their fractions [4,30]. Downstream processing of protein hydrolysates by a properly designed UF cascade and recycling loops is able to refine several bioactive peptide fractions at once, possibly improving their functional/biological activity by increasing peptide purity [3,29].

The first step of protein and peptide fractionation involves the use of higher MWCO UF membranes (500–50 kg mol^−1^), intended to reject intact/non-extracted proteins, fibers, polysaccharides and other macromolecules that were not removed in previous steps. One or more fractionation steps can be performed in this MWCO range. High molecular weight peptides with emulsifying and stabilizing activities may be recovered in the first retentate fractions, as shown in Figure 2. The last permeate from the fractionation cascade at 500–50 kg mol^−1^ goes on for further fractionation (UF membranes) of MWCO of 50–1 kg mol^−1^, in order to recover bioactive peptides and amino acids [3] (Figure 2).

Fractions of interest (permeate or retentate) from the fractionation cascade may be purified (decrease impurity concentration) and peptides can be even isolated, either due to application requirements (e.g., for pharmaceutical industry use) or for analytical purposes. Depending on the peptide mixture properties, peptides are isolated thanks to techniques based mainly on sieving (size-exclusion chromatography, low MWCO UF, NF), charge-based techniques (pH-induced precipitation, electrodialysis, ion-exchange chromatography—as discussed in Section 4), techniques that detect differences in hydrophobic interactions (solvent precipitation, reversed-phase chromatography) or even affinity and special attribute molecular methods (affinity chromatography, immunoaffinity). Although chromatographic methods are mainly used for analytical purposes, the NF membrane technique is one of the industrial processes most frequently used to purify low molecular weight peptides, employing membranes in the range of 100–1000 g mol^−1^ [3,4].

The concentration step of the resulting streams is not mandatory but, depending on the end-application of the ingredients, it is a recommended procedure. The use of bioactive peptides or other isolated protein fractions is sometimes not practicable in small concentrations. Thermal or chemical processes such as dehydration, rotary or vacuum evaporation and chemical precipitation might be used but they are employed less and less because most bioactive peptides are thermolabile and may be denatured while losing activity in the presence of chemicals and solvents. Instead, NF, reverse osmosis and spray-drying are preferred technologies because they consume a smaller amount of chemicals and energy [3,29]. Spray-drying may be successfully used to protect bioactive peptides and extend their shelf-life when appropriate process parameters are employed and the characteristics of each matrix are taken into account [29,31,32].

In sum, the design of an efficient fractionation process for peptide mixtures from complex by-products requires knowledge of: (1) target peptide or protein fraction properties (amino acid sequence, mass ratio, isoelectric point, hydrophobicity, bioactive properties), (2) rigorous characterization of feed composition and sensitivity of feed components to processing conditions and (3) presence of main contaminants that may need to be separated from the target protein fractions [3].

## 3. Membrane Fractionation of Protein Hydrolysates: Challenges, Limitations, Advantages and Solutions

Membrane separation processes offer several advantages: mild operating conditions without any state changes, low energy requirements in comparison to conventional concentration processes, high selectivity, wide range of applications, modular design, simplicity in continuous operation, integration and scaling up [2,4,25]. In the context of protein hydrolysate separation, membrane processes are able to maintain protein stability throughout the process, and a high-resolution separation is possible at ambient/low temperatures. They do not require the use of solvents and other chemicals [2,30]. Indeed, several biotechnological and pharmaceutical applications count on those advantages to obtain high-resolution fractions [4,5].

Membrane and process operations to conduct bio-separations may involve high costs, especially for establishing the installed membrane area (e.g., the price of new membranes and their modules). However, the value added to the recovered product is usually more significant. An increase of 8-10% in sales of membranes and modules for food processing and water treatment applications may lead to a gradual decrease of membrane technology costs [1,2].

Main limitations of the separation of protein-rich products by membranes are fouling and limited selectivity. Proteins are easy foulants because they have a complex molecular structure and multiple charged groups that readily interact with the membrane surface, water and ions, with the latter affecting their real size and solubility [3,33]. Fouling control strategies are indispensable to maintain acceptable flux levels and ensure microbiologically safe membrane operations, minimizing the growth of microorganisms and the formation of biofilms [2].

Several solutions are being investigated to improve mass transfer and limit fouling formation. Hydrodynamic management strategies include the use of different modules that ensure operation at turbulent regimes. Intelligent membrane cleaning methods are becoming more efficient with deeper knowledge of foulant complexity and composition. Operations such as back flushing and pulsing, and use of non-conventional technologies (pulsed electric fields and ultrasound) have been reported to improve cleaning and even mass transport in UF [1]. The application of electric fields with NaCl was able to completely clean a zirconium dioxide/titanium dioxide UF membrane of 15 kg mol^−1^ MWCO fouled with whey model solutions [34]. Ultrasound technology has been used to enhance permeate flux. Higher US frequencies (100–1000 kHz) form smaller bubbles that culminate in the creation of several focus of increased temperature. At frequencies of 20–40 kHz, collapses are created by large bubbles that result in shockwaves that increase turbulence. These characteristics are probably related to the effective use of US in the range of 20–50 kHz in membrane processes, but the mechanisms involved in flux enhancing and the cleaning effects of ultrasound are yet not clear (higher turbulence vs. sonication effects) [35]. US showed up to 20% and 40% enhancement, respectively, for 1 MHz at 0.28 m s^−1^ and 1.5 m s^−1^ crossflow velocity (whey UF at a transmembrane pressure smaller than 1.5 bar) [36]. The development of new engineered membrane materials is also a prominent field in membrane technology. Surface modification is aimed at improving membrane resistance to protein adsorption and to increase permeation of hydrophobic membranes. The increase of surface hydrophilicity can effectively minimize protein adsorption, improve membrane permeability and prevent membrane fouling [2,5].

The understanding of critical flux and fouling phenomena can be used as a strategy to maintain high selectivity and mass throughput of UF and NF operations. The critical flux has been used as the point in a flux vs. transmembrane pressure at which this line becomes non-linear and as the point where fouling appears [37]. The critical flux calculation can be described by Darcy’s resistance-in-series model [38,39], presented in Equation (1). In this equation are presented the flux of permeate (Jp, m^3^ m^−2^ s^−1^), the dynamic viscosity of the solution (μ, Pa · s), the operating transmembrane pressure (ΔP, Pa) and the resistances on the feed side of the boundary layer (*R*, m^−1^).

(1) $$Jp=\frac{\Delta P}{\mu \;R}$$

Equation (2) described the flux equation when the flux value is below the critical value. For the strong forms of critical flux, the only resistance is that of the membrane ($R_{m}$), whereas for the weak form, the resistance caused by the adsorption of molecules onto the membranes surface ($R_{ads}$) is added. Equation (3) presents the mathematical relation when the flux is above the critical flux, when at least the reversible ($R_{rev}$) or irreversible ($R_{irrev}$) fouling are non-zero. Again, for the weak form of critical flux, the $R_{ads}$ is accounted for.

(2) $$Jp=\frac{\Delta P}{\mu \;R_{m}\;\left(+R_{ads}\right)}$$

(3) $$Jp=\frac{\Delta P}{\mu \;R_{m}+R_{rev}+R_{irrev}\;\left(+R_{ads}\right)}$$

Working within the limits of the critical flux and the pressure control region (low pressures, low volumetric reduction factors and feed concentrations) can reduce fouling and increase separation performance. After a threshold concentration of solutes on the membrane surface (the mass transfer-controlled region), higher productivity can only be achieved if there is an increase in the mass transfer coefficient [3,5,40].

The detailed investigation of mass transfer and thorough description of fouling mechanisms through theoretical and modelling studies have provided fundamental insights that are imperative to further improvements in membrane performance. So far, no model is universally applicable or satisfactory, but as simulation evolves, phenomena description and understanding, as well as technology maturity, also evolve. Recently, there has been a multi-objective optimization of design and operational conditions to maximize product yield and purity for fractionation of fish by-product protein hydrolysates using UF and NF [41]. The proposed modelling strategy included economic and environmental aspects in the optimization of technical objectives. They have observed that when the minimization of total costs was included as an objective, the performance of the process was affected, mainly the yield, which was considerably reduced. The consumption of fresh water (that typically represents an important part of operational costs), did not influence maximal product purity or the process yield. In other words, water consumption could be minimized, but the viscosity of the solutions and the associated risk of membrane clogging were cited as important factors to be considered to ensure adequate processing [41].

In sum, we have presented some of the efficient solutions to reduce fouling and increase selectivity and productivity of membrane processes that are being developed [30,42].

## 4. Peptide Separation and Purification by Charge

The separation of peptides and protein fractions depend on their charge and interactions. In this context, charge-based membrane separations can be used as a next separation step after UF or as another separation strategy, depending on the envisaged separation outcome. Charge-based membrane separations depend on the conductivity of feed streams, which are usually low in food and biotechnology streams. Some of these processes (e.g., electrodialysis or electrophoresis) have higher energy requirements than conventional membrane processes, and heat may be produced, depending on the operating conditions, which could result in the degradation of molecules. Despite those limitations, charge-based membrane separation processes are aligned with the researchers’ efforts to improve peptide refining and can replace chromatography, which is mostly used for analytical purposes [29]. Several simultaneous size and charge-based separation techniques are available, including high-performance tangential flow filtration (HPTFF), electrophoretic membrane contactor processes, membrane chromatography, electrically enhanced membrane filtration (EMF), UF using charged membranes, electro-ultrafiltration using pulsed fields, electrodialysis and electrodialysis combined with UF membranes (EDUF) [1,3,4]. Among them, one of the most promising techniques recently used for peptide separation is electrodialysis and EDUF.

Electrodialysis is a membrane technology involving ion-exchange membranes and an electrical potential as driving force. Species mobility depends not only on their electrophoretic mobility but also on sieving effects (as is the case of EDUF). The extent of transferred mass flow depends on the electrophoretic mobility of peptides, on the presence of other charged components in the feed, on the solution’s pH, and on the nature of membranes used in the process (dense or porous membranes). Because each species migrates at a specific rate, depending on the configuration previously selected, several outlet streams can be obtained simultaneously, and each of them is either depleted or enriched in a specific peptide or species [4,43]. When porous membranes are employed in EDUF, they act as electrophoretic membrane contactors, in which separation occurs while taking into account charge and molecular weight peptide differences. This technology has been reported as a very selective one, capable of separating targeted peptides from complex mixtures and residues from food and agro by-products [44,45,46]. The simultaneous separation of anionic and cationic peptides from a herring milt hydrolysate by EDUF using UF membranes of 50 and 20 kg mol^−1^ MWCO was reported [45]. Peptide migration rates through the first membrane were 44- and 20-fold higher for anionic and cationic peptides, respectively, in comparison to the second membrane. EDUF at pH 7.0 allowed the separation of peptides with positively charged arginine and lysine in the cationic recovery compartments and peptides with negatively charged asparagine and glutamine in the anionic recovery fractions. Durand et al. [45] found that the anionic fraction obtained after the 50 kg mol^−1^ MWCO membrane had the highest antioxidant activity whereas anti-inflammatory activities were higher in the cationic fractions collected after the first and second membranes. EDUF using 20 kg mol^−1^ MWCO UF membranes enabled the separation of arginine-containing peptides in a defatted flaxseed protein hydrolysate, enhancing hypotension effects in vivo of fractions, since these effects have been associated with the presence of arginine in active peptides [47]. EDUF has recently been considered for large scale peptide production in substitution to several chromatographic operations. EDUF was exploited as a tool to ease the isolation of antihypertensive peptides from a protein hydrolysate of rapeseed protein isolate. An anionic peptide fraction with 44% negatively charged amino acids and a cationic peptide fraction with 28% positively charged amino acids were recovered after 6 h of operation in an EDUF process that employed 20 kg mol^−1^ MWCO membranes. At a feed concentration of 1.7 mg of peptides per mL, the system could operate for 18 h without any indication of membrane fouling [48]. The sustainable aspect of EDUF was explored in the valorization of the bovine cruor, i.e., the red cells fraction of the blood by-product from slaughterhouse processing. A positively charged antimicrobial peptide, obtained from hemoglobin, was enriched 24-fold using a 10 kg mol^−1^ MWCO membrane [44]. Associated costs of producing peptides from EDUF technologies was reported to range from 0.3 to 0.5 Canadian dollars per gram of peptides for an effective filtration area of 10 m^2^ [47]. These results indicate the potential of this technology in the field of recovery of peptides from by-products, but competitive evaluation was not done because the cost of peptide recovery using other separation techniques was not yet reported to our best knowledge (e.g., ultrafiltration or chromatography). Higher migration rates and production at larger scale are some of the perspectives of EDUF, in comparison to chromatography and membrane chromatography techniques. However, these perspectives face three major challenges: understanding the underlying mechanisms of transport in complex matrices, finding ways to enhance peptide migration while keeping quality and reducing the high costs of this technology.

Membrane chromatography is also a promising technology in the treatment of protein hydrolysates. In this technology, membranes act as contactors, and chromatographic properties such as ion-exchange, affinity or reversed-phase, are transferred to the membranes [4]. Membranes for membrane chromatography are produced by grafting specific ligands onto the membrane surface (affinity chromatography properties), embedding the surface with ion-exchange resins or groups that are added via surface modification techniques (ion-exchange and reversed-phase chromatography properties). Targeted components or biomolecules are adsorbed to these functional groups or ligands during the flow. This technology has great potential to process design. Different membrane porosities and membrane pore sizes can be used, which could influence the access of molecules to the ligands. Process conditions can also affect transport and separation, with the efficiency of membrane chromatography tightly linked to membrane module design [49]. Membrane chromatography is a very versatile process, developed to overcome the main drawbacks of packed-bed columns, in which the transport is diffusion limited and pressure drops are very high [50]. High operational costs, difficulties with column packing and scaling up have also driven the research on the field. The main advantage of membrane chromatography is the improved mass transfer, as the transport through the porous membrane structure reduces the limitations linked to the diffusion in beads [49]. Despite these advantages, for analytical purposes, membrane chromatography techniques are still not as used as conventional chromatographic techniques [4,5,33].

## 5. Membrane Fractionation and Purification of Yeast Protein Hydrolysates: Recovery of Bioactive Peptides

### 5.1. Challenges Involving Yeast-Product Separation by Membranes

**Figure 3:**
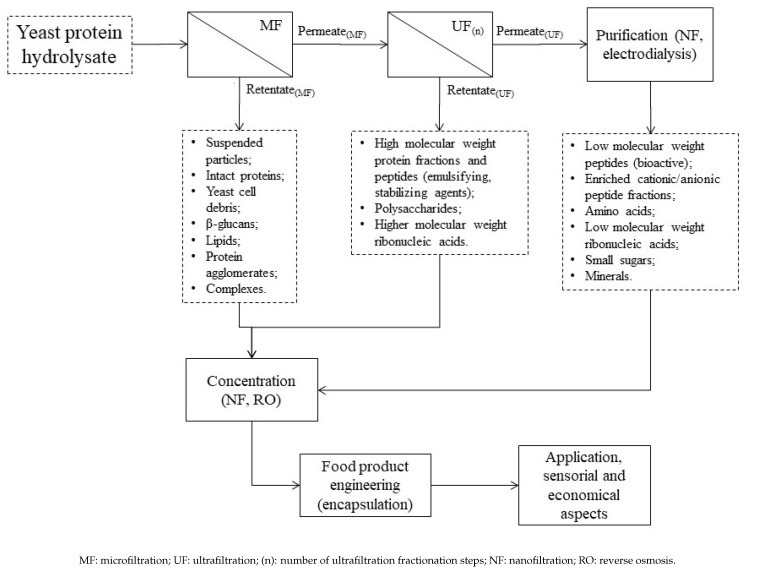
Use of membrane separation technology in the recovery of protein-rich ingredients and bioactive peptides from yeast protein hydrolysates and the main compounds recovered in fractions.

Figure 3 shows how some membrane technologies can be used in the recovery of protein-rich fractions and peptides from yeast protein hydrolysates and which components are involved during processing. Cultivated and spent yeast protein hydrolysates are very complex matrices after disruption and enzymatic hydrolysis. Stabilization unit operations or pre-treatments (Figure 1) are usually capable of removing most of the high molecular weight compounds that may disturb protein and peptide fractionation performance, such as cell debris, non-hydrolyzed proteins and other non-protein components (Section 2). These components are reported to decrease the resolution of analytical techniques if they are not removed prior to chromatography and electrophoresis of spent brewer’s yeast materials [9].

Feed composition is one of the important factors to be considered in the design of an efficient separation process. Table 1 shows the composition of macronutrients of SBY protein hydrolysates produced via enzymatic processes. Proteins constitute the main compound present, followed by polysaccharides. Smaller amounts of ashes, lipids, minerals and vitamins are also found. Ribonucleic acids are a key component of yeast products because they can limit product consumption if they are found in high amounts in the end-product (>3%, d.w.). In humans, nucleic acids are metabolized to uric acid, which can be involved in health conditions such as kidney stones formation or gout [51].

During peptide fractionation, the main reported foulants are protein fragments (which, depending on the medium conditions, may form aggregates or complexes with ribonucleic acids (RNA) or polysaccharides), fibers such as β-glucans and other polysaccharides complexes [16,51,54]. The recovery of β-glucans might take place before peptide fractionation, because of the difference of molecular weight range of those molecules. UF conditions using 100 kg mol^−1^ MWCO membranes were optimized to recover high molecular weight β-glucans from oat mill waste [55]. This strategy was still not reported for SBY or other spent yeasts (from sugarcane and distilleries) but membrane technologies (MF and 10–100 kg mol^−1^ UF MWCO membranes) have been successfully employed in the recovery of β-glucans from cereals, algae and mushrooms [56,57].

The pI of yeast proteins is around 4–5 [51], but there are only a few studies to date on the effect of pH in the separation of spent yeast hydrolysates [54]. The influence of feed pH on the rejection of proteins during UF and NF illustrate the existence of charge effects on separation performance. Peptides have ionizable groups in the molecule structure in the N-terminal and C-terminal residues as well as in the side chains of amino acids that form them. Thus, the pH value of the feed solution influences the charge of those groups and the electrostatic interactions between molecules and the membrane [3,21]. She et al. [58] found that the rate and extent of flux decline as well as fouling intensity are augmented around the pI for the UF of bovine serum albumin solutions. Apart from the pI, separations undertaken at high pH values (8.0) usually present higher peptide transmission in comparison to the regions close to the pI because of higher repulsion interactions that prevent fouling. Saidi et al. [21] detected a three-fold higher permeate flux when the UF of a tuna protein hydrolysate was performed at pH 8 in comparison to pH 3. They also found that the retention rate increased as the pH value increased from 3 to 5, but it was kept constant for higher pH values. At a defined pH value of the feed, the separation of specific classes of peptides may be favored. At basic pH values, a very high rejection of acidic peptides was achieved in a membrane with negative net charges at this pH [59]. In sum, the observed effect of the pH value on membrane performance is modulated not only by protein charge and conformation, but is also represented by the charges of the membrane under these conditions, as well as the charges of the compounds that constitute the polarized layer [3]. Higher permeate fluxes are usually found for pH values far from the pI.

Salt content in protein hydrolysates is not often determined because the concentrations are usually not high compared to the other components. On the other hand, in yeast hydrolysates salt content may be important depending on hydrolysate processing conditions, because during autolysis, salts can be added to increase the extent of cell rupture [51,60]. A high concentration of salts increases the ionic strength of the solution which in turn may alter both (1) the solubility of proteins (the higher the ionic strength, the smaller the activity coefficients of charged species, and thus smaller is proteins solubility) and (2) mass transport of proteins during membrane operations (caused by changes in electrostatic interactions between solutes and/or the membrane). These effects are attributed to a higher concentration of salt ions near the membrane surface, which adds up resistance to mass transfer [61]. A recent investigation, which monitored fouling formation in the separation of bovine serum albumin with controlled ionic strength, showed that the influence of this parameter on flux and extent of fouling depends on stage of filtration. In the initial stages of UF, membrane fouling rate decreases with increased ionic strength, probably because of increased repulsion forces. With a longer operation time, a flux decline rate at 100 mM is superior to the conditions of low or no ionic strength. A higher concentration of bovine serum albumin adjacent to the membrane surface confirmed the higher degree of fouling as the UF was carried out [61]. The determination of proper ionic strength conditions is complex because this implies knowledge of the characteristics of membrane charge and surface properties as well. Changes in ionic strength of the bulk solution are able to reduce the fouling extent by controlling those interactions, depending on the pH. In general, high ionic strength feed streams caused reduced selectivity and throughput of UF; this may be due to the fouling caused by the compression of the electrical double layer of foulants deposed on the membrane surface or within the pores [58]. Particular attention must be paid to these physico-chemical parameters (pH, ionic strength) of the hydrolysate solution in order to develop an adequate separation strategy capable of fractionating proteins from SBY.

The use of yeast as a food ingredient can be limited by its high content of RNA, which is often extracted with protein molecules by conventional methods [51]. Processing strategies to promote the decrease of RNA content in yeast products are usually limited to the extraction step. Chemical methods are used to precipitate ribonucleic acids, but important amounts of proteins are precipitated as well. Differences in RNA and protein structure and their charge suggest that the separation of RNA from protein fractions in spent brewer yeast protein hydrolysates could be performed by using membrane separation technologies. Recently, diluted torula yeast (mixture of heterogeneous RNA) solutions have been separated in polyethersulfone and regenerated cellulose UF membranes. Experiments were made in amicon dead-end filtration cells of 4.1 cm^2^ of effective filtration area, at room temperature and low pressure (0.06 to 0.90 bar). Adsorption of RNA in regenerated cellulose membranes was significant but this effect was minimal in polyethersulfone. In polyethersulfone membranes of 300 kg mol^−1^ MWCO, 95% of RNA was permeated, while 50 and 100 kg mol^−1^ MWCO membranes were able to reject most of the RNA at low flux [62]. In another study, Manzano et al. [63] evaluated synthesized RNA transmission through polyethersulfone UF membranes of 50, 100 and 300 kg mol^−1^ MWCO. RNA structure (hairpin or linear, with an equivalent number of nucleotides) affected the extent of transmission. This finding was attributed to the effective size of the molecule, which depends on the molecule structure. RNA transmission at pH 7.5 was enhanced upon the addition of NaCl (100 mM), which is believed to affect molecule size and increase the ionic strength of the medium, causing electrostatic repulsion between the negatively charged polyethersulfone membrane and negatively charged RNA. Additional studies are required to explain RNA transmission mechanisms and to explore RNA separation in more complex matrices, such as in yeast extracts and protein hydrolysates of SBY.

In sum, there are various challenges involving membrane separation of yeast-based by-products such as SBY. The complex composition suggests that the choice and operating conditions of the membrane separation process should follow precise separation objectives. The selection of the pre-treatment process, membrane, module, feed conditions (pH, concentration, ionic strength) and operational conditions is decisive to guarantee membrane process performance of spent yeasts protein hydrolysates.

Several streams may be produced during the recovery of target compounds, and valorization of these streams needs to be maximized as much as possible. The development of sustainable processes that take into account all the generated fractions and all spheres of the process is extremely important to maintain the environmental and economic viability of the technology, especially when the transformation of by-products is envisaged.

### 5.2. Strategy of Fractionation of SBY and Yeast Protein Hydrolysates

Table 2 and Table 3 show the state-of-the-art of fractionation and concentration of protein hydrolysates from cultivated *Saccharomyces* sp. cells, spent yeasts from distilleries, sugar cane processing and brewing (SBY).

The concentration step of SBY proteins and polysaccharides was carried out with MF and UF polyethersulfone membranes (0.2 m and 5 kg mol^−1^ MWCO) and yield of about 95% for both proteins and polysaccharides was obtained. The influence of yeast extract concentration, feed pH and operating pressure on the yield of polysaccharides and protein nitrogen was investigated using response surface methodology (Box-Behnken design). Optimized separation conditions were determined at the center of the study ranges: 2.7% (m/m) feed concentration at pH 5.0 and 0.97 bar of operating pressure, but the study did not explain why these conditions were the optimal ones concerning mass transfer and SBY characteristics [54]. In several studies of another research group, UF membranes of 30 kg mol^−1^ and 10 kg mol^−1^ MWCO were used to fractionate a protein hydrolysate from cultivated *S. cerevisiae* before the freeze-dried yeast extracts were used in in vitro and in vivo determinations of anti-obesogenic and anti-stress activities of yeast peptides [64,65,66,67,68,69,70].

With the intent of creating innovative ingredients rich in polysaccharide and protein from SBY, series of ultra and nanofiltrations before and after hydrolysis (10 and 3 kg mol^−1^ MWCO) were performed in a pilot system, resulting in four fractions. Proteins were mainly present in the higher molecular weight fractions while polysaccharides were mostly represented by simple sugars released by the autolysis process, in smaller molecular weight fractions. Minerals were fractionated as well: sodium concentration in the most concentrated fraction differed from 4- to 24-fold to others. Free amino acid profile was also changed by UF, thus indicating that the fractionation process can be developed to refine specific free amino acids. SBY amino acids such as glutamine, glutamic acid, arginine, alanine, tyrosine and valine were enriched in permeates of 3 kg mol^−1^ MWCO membranes [16].

Notably, UF (MWCO of 30-3 kg mol^−1^) is one of the main fractionation tools applied for the separation of yeast protein hydrolysates [9,10,16,23,54,64,65,66,67,68,69,70,71,72,73,74,75]. The application of membrane technology for those products was reported at different scales (from the use of UF cartridges and amicon cells [9,10,23,64,65,66,67,68,69,70,71,72,73,74] to pilot-scale systems with a highly effective permeation area [16,54,75]). Chromatographic techniques based on sieving (Size-Exclusion Chromatography—SEC) and based on hydrophobicity of peptides (Reversed-Phase High-Performance Liquid Chromatography—RP-HPLC or Reversed-Phase Solid Phase Extraction—RP-SPE) are also very common to promote the fractionation of yeast protein hydrolysate for analytical purposes [10,13,16,71,73,74,75,76]. A protein hydrolysate of cultivated *Saccharomyces cerevisiae* cell produced by using trypsin was fractionated by capillary reversed-phase liquid chromatography (RPLC) for the evaluation of yeast proteome as, a two-dimensional proteomics separation approach. [14]. Jung et al. [23] studied several processes to concentrate SBY hydrolysates (acid precipitation, activated carbon, UF and a combination of these) and reported a better performance with UF (10 kg mol^−1^ MWCO), allowing for a 20-fold concentration of a specific peptide (Cyclo-His-Pro) in comparison to its initial concentration. As shown in Table 2 and Table 3, concentration of yeast hydrolysates is mainly performed by freeze-drying, but NF, RO—as well as conventional techniques (vacuum evaporation and precipitation)—have also been reported.

**Table 2:** Fractionation of yeast (*Saccharomyces* sp.) protein hydrolysates by membrane technologies and/or chromatography in the production of bioactive peptides.

Yeast	Fractionation/Concentration	Peptide Analytical Techniques	Purpose	References
Sugarcane spent yeast (*S. cerevisiae*)	Fractionation: UF cartridges (5 kg mol^−1^ MWCO) and FPLC with IMAC-Fe(III) resin chromatography. Concentration by freeze-drying.	-	Iron-chelating peptides	de la Hoz et al. [71]
Cultivated yeast (*S. cerevisiae*)	Fractionation: UF cartridges (10 and 30 kg mol^−1^ MWCO). Concentration by freeze-drying.	-	Peptides with anti-obesogenic and anti-stress properties	Jung et al. [64], Park et al. [65], Jung et al. [66], Kim et al. [67], Lee et al. [68], Lee et al. [69], Kim et al. [70], Kim et al. [72]
Cultivated yeast (*S. cerevisiae*)	Fractionation: UF (10, 5 and 3 kg mol^−1^ MWCO) and RP-HPLC (C_18_ column). Concentration: freeze-drying.	MS (MALDI-TOF-TOF) for peptide sequencing	Peptides with antioxidant activity	Mirzaei et al. [73]
Cultivated yeast (*S. cerevisiae*)	Fractionation: Dialysis (6–8 kg mol^−1^ MWCO) and RP-SPE cartridges (C_18_). Concentration: vacuum evaporation.	RP-HPLC (C_18_ column)	Glyco-peptide with anti-inflammatory activity	Williams et al. [76]
Cultivated yeast (*S. cerevisiae* K-7)	Fractionation: labscale UF (5 kg mol^−1^ MWCO); SEC (Sephadex G-25) and RP-HPLC (C_18_ column). Concentration: freeze-drying	LC-MS (peptide sequencing)	Anti-angiogenic peptides	Jeong et al. [74]

ACE-I: inhibitory activity of the angiotensin-converting enzyme; FPLC: Fast Protein Liquid Chromatography; HPLC: high-performance liquid chromatography; IMAC: Immobilized metal affinity chromatography; LC: liquid chromatography; MALDI: Matrix assisted laser desorption/ionization; MS: mass spectrometry; MWCO: molecular weight cut-off; RP: reversed-phase; SEC: size-exclusion chromatography; SPE: solid phase extraction; TOF: Time-of-Flight; UF: ultrafiltration.

**Table 3:** Fractionation of protein hydrolysates from spent yeasts from brewing by membrane technologies and/or chromatography in the obtention of bioactive peptides.

Yeast	Fractionation/Concentration	Peptide Analytical Techniques	Purpose	References
SBY	Fractionation: adsorbing column (Amberlite XAD-2 resin), SEC (Sephadex G-25), RP-HPLC (C_30_ column). Purification: gel filtration phase HPLC (Diol column)	LC/MS/MS (amino acid sequencing)	Peptides with ACE-I activity	Kanauchi et al. [13]
SBY	Fractionation: UF (10 kg mol^−1^ MWCO). Concentration: acid precipitation, activated carbon adsorption.	HPLC (peptide profile)	Antioxidant and anti-diabetic peptides	Jung et al. [23]
SBY	Fractionation: UF module of effective permeation area of 7.4 m^2^ (10 and 3 kg mol^−1^ MWCO). Concentration: Reverse osmosis and freeze-drying.	RP-HPLC (C_18_ column); MS (MALDI-TOF-TOF for amino acid sequencing)	Nutritional ingredient rich in protein and polysaccharides ^1^; peptides with antioxidant and ACE-I properties ^2^	Amorim et al. [16] ^1^; Amorim et al. [75] ^2^
SBY (*S. pastorianus*)	Fractionation: NF in amicon cell (3 kg mol^−1^ MWCO). Concentration: freeze-drying.	SEC (Superdex 200 and Superdex peptide 10/300GL)	Peptides with ACE-I activity	Amorim et al. [10]
SBY	Fractionation: MF and UF in hollow fibers with effective permeation area of 0.05 m^2^ (0.2 m and 10 kg mol^−1^ MWCO).	-	Polysaccharide and protein-rich fractions	Huang et al. [54]
SBY (*S. pastorianus*)	Fractionation: UF in flat sheet module of effective permeation area of 0.0016 m^2^ (30 and 10 kg mol^−1^ MWCO).	Electrophoresis (SDS-PAGE)	Antioxidant peptides	Marson et al. [9]

ACE-I: inhibitory activity of the angiotensin-converting enzyme; HPLC: high-performance liquid chromatography; LC: liquid chromatography; MALDI: Matrix assisted laser desorption/ionization; MF: microfiltration; MS: mass spectrometry; MWCO: molecular weight cut-off; NF: nanofiltration; RP: reversed-phase; SDS-PAGE: sodium dodecyl sulfate–polyacrylamide gel electrophoresis; SEC: size-exclusion chromatography; SBY: spent brewer’s yeast; TOF: Time-of-Flight; UF: ultrafiltration. ^1^ [16]. ^2^ [75].

To summarize, the use of UF and NF in the fractionation and concentration of peptides and other components of yeast is incipient, but is gradually increasing. The use of membranes in the range of 3–30 kg mol^−1^ MWCO seems to be adapted to the separation of yeast protein fractions and peptides. Polysaccharides appear to be one the main compounds separated along with proteins, but new studies should also take into consideration the separation of yeast peptides from ribonucleic acids, which are reported to limit application of yeast-based products, and minerals, which can be in higher amounts when compared to other protein hydrolysates. Membrane technology application in the downstream of yeast by-products requires more deep studies to comprehend the transport phenomena and to define ways to improve it. The impact and choice of operating parameters is still not clear and requires further understanding. For spent yeasts, membrane processes are still mainly used as an analytical tool, and the separation process is not explored in terms of process parameters. Mass transfer, concentration polarization and fouling phenomena, effect of feed characteristics (composition, pH, ionic strength, concentration) and interactions between the membrane material and SBY hydrolysate are scarcely reported in the literature but these studies could definitely improve separation performance.

Operational costs are also a very relevant concern in the processing of by-products and waste streams with spent yeasts [1,8]. The separation and fractionation of yeast-based protein hydrolysates in fewer steps or in higher concentrations is desired, for economic reasons. In this context, production of yeast protein hydrolysates using combined technologies such as membrane reactors could be a possibility to be explored as well. Further studies focused on the comprehension of fouling phenomena and mass transfer mechanisms are still needed to improve and extend the scope of membrane technology for an efficient and cost-effective production of peptides from cultivated and spent yeasts.

## 6. Conclusions

The coherent application of membrane separation technologies on the recovery of value-added compounds such as bioactive peptides from agro-industrial and biotechnological by-products depends on the development of integrated processes adapted to the specificities of those materials. High-resolution fractions can be obtained if a well-designed strategy is chosen - from by-product extraction to downstream processing and product engineering.

Peptide and protein fractionation should take into account the associated effects of feed, membrane and processing parameters so that maximum membrane performance is achieved, and an economically viable recovery of peptides is possible. Integration of sieving and charge-based membrane techniques as well as the elucidation of underlying mechanisms of separation may improve the throughput of the technology, which is much required in bio-separations.

Several possibilities involve the recovery of value-added compounds from cultivated and spent yeasts. The production of fractions with high peptide content but with low content of RNA, polysaccharides and fibers poses a great challenge. The recovery of various fractions enriched in different high-added-value components such as β-glucans, peptides for different applications, oligosaccharides, minerals and amino acids is possible through multiple fractionation processes and may increase the economic viability of yeast by-product processing.

## Figures and Tables

**Table 1:** Macronutrients composition in spent brewer’s yeast protein hydrolysates produced using enzymatic hydrolysis.

Macronutrients (g 100 g^−1^, d.w.)	SBY Enzymatic Hydrolysate [9,12,16,52,53]
Total nitrogen	1.5–12.0
Protein nitrogen	9.3–69.0
Free amino nitrogen	28–35
Ribonucleic acids	5.6
Total sugars	3.0–48
Lipids	0.2–1.0
Ashes	3.0–22.0

All composition data are expressed in dry weight (d.w.). Because protein content is determined using various analytical techniques and calculations, we presented only protein nitrogen data (considering original conversion factors) and the corresponding total nitrogen content.

## Data Availability

Data sharing not applicable.

## Abbreviations

The following abbreviations are used in this manuscript:

ACE-I	Inhibitory activity of the angiotensin-converting enzyme
EDUF	Electrodialysis using ultrafiltration membranes
EMF	Electrically enhanced membrane filtration
FPLC	Fast Protein Liquid Chromatography
HPLC	High-performance liquid chromatography
HPTFF	High-performance tangential flow filtration
IMAC	Immobilized metal affinity chromatography
LC	Liquid chromatography
MALDI	Matrix-assisted laser desorption/ionization
MF	Microfiltration
MS	Mass spectrometry
MW	Molecular weight
MWCO	Molecular weight cut-off
NF	Nanofiltration
pI	Isoelectric point
RNA	Ribonucleic acids
RO	Reverse osmosis
RP	Reversed-phase
SBY	Spent brewer’s yeast
SDS-PAGE	Sodium dodecyl sulfate–polyacrylamide gel electrophoresis
SEC	Size-exclusion chromatography
SPE	Solid phase extraction
TOF	Time-of-Flight
UF	Ultrafiltration
